# Prediction of major bleeding in cirrhotic ICU patients using the dic score and coagulation parameters

**DOI:** 10.1186/2197-425X-3-S1-A972

**Published:** 2015-10-01

**Authors:** A Drolz, T Horvatits, K Roedl, K Rutter, N Kneidinger, C Bopp, R Wüstenberg, C Zauner, G Heinz, P Schellongowski, T Perkmann, M Trauner, V Fuhrmann

**Affiliations:** Intensive Care Medicine, University Medical Center Hamburg-Eppendorf, Hamburg, Germany; Medical University of Vienna, Vienna, Austria

## Introduction

The disseminated intravascular coagulation (DIC) score is a predictor of outcome in critically ill patients [1]. Yet, disturbances of coagulation are a common finding in patients with liver cirrhosis. Thus, the prognostic value of the DIC score and its subcomponents in patients with liver cirrhosis is unclear.

## Objectives

Aim of this study was to evaluate the predictive abilities of the DIC score and routine coagulation parameters regarding major bleeding and outcome in patients with liver cirrhosis at the ICU.

## Methods

A total of 1493 ICU patients were studied prospectively. Laboratory analysis including routine coagulation parameters was conducted on on a daily basis during the ICU stay. New onset of major bleeding during the ICU stay was documented. Mortality was assessed on site or by contacting the patients or the attending physician.

## Results

Two hundred eleven ICU patients had liver cirrhosis (12%). Admission- and peak DIC score within 48 hours from ICU admission differed significantly between patient with and without cirrhosis. Significant differences between patients with and without cirrhosis were observed in platelet count (PLT), fibrinogen and prothrombin time (PT), activated Partial Thromboplastin Time (aPTT) and d-dimer.

In patients with liver cirrhosis, DIC score was associated with major bleeding at the ICU (AUROC 0.645, p < 0.01); however, this was mainly attributable to the subscores for fibrinogen and PLT. Fibrinogen (AUROC 0.749, p < 0.001), PLT (AUROC 0.651, p < 0.01) and aPTT (AUROC 0.640, p < 0.01) on admission were associated with major bleeding in cirrhotic ICU patients. Additionally, bleeding on admission was identified as a risk factor for new onset of bleeding in these patients (OR = 17.6 (95% CI 7.0-44.1), p < 0.001). Fibrinogen level < 60 mg/dl, PLT < 30 G/l and aPTT > 100 s were identified as highly specific thresholds for prediction of major bleeding in cirrhosis. These three criteria remained significantly associated with major bleeding after correction for age, sex, admission SOFA score and bleeding on admission.

Major bleeding in cirrhotic patients was associated with poor outcome. One-year-mortality in cirrhotic patients with and without major bleeding was 89% and 68%, respectively (p < 0.05 between groups).

## Conclusions

Abnormal coagulation parameters and increased DIC scores are found in the majority of patients with liver cirrhosis at the ICU, and they correspond to increased bleeding risk. Fibrinogen, PLT and aPTT were identified as useful tools for prediction of major bleeding in cirrhosis.
